# Applications of conceptual models from lifecourse epidemiology in ecology and evolutionary biology

**DOI:** 10.1098/rsbl.2022.0194

**Published:** 2022-07-20

**Authors:** Zachary M. Laubach, Kay E. Holekamp, Izzuddin M. Aris, Natalie Slopen, Wei Perng

**Affiliations:** ^1^ Department of Ecology and Evolutionary Biology (EEB), University of Colorado Boulder, Boulder, CO, USA; ^2^ Mara Hyena Project, Karen, Nairobi, Kenya; ^3^ Department of Integrative Biology, Michigan State University, East Lansing, MI, USA; ^4^ Division of Chronic Disease Research Across the Lifecourse, Department of Population Medicine, Harvard Medical School and Harvard Pilgrim Health Care Institute, Boston, MA, USA; ^5^ Department of Social and Behavioral Sciences, Harvard T.H. Chan School of Public Health, Boston, MA, USA; ^6^ Department of Epidemiology, Colorado School of Public Health, University of Colorado, Aurora, CO, USA; ^7^ Lifecourse Epidemiology of Adiposity and Diabetes (LEAD) Center, University of Colorado, Aurora, CO, USA

**Keywords:** conceptual model, ecology and evolutionary biology, causal inference, epidemiology, lifecourse, developmental plasticity

## Abstract

In ecology and evolutionary biology (EEB), the study of developmental plasticity seeks to understand ontogenetic processes underlying the phenotypes upon which natural selection acts. A central challenge to this inquiry is ascertaining a causal effect of the exposure on the manifestation of later-life phenotype due to the time elapsed between the two events. The exposure is a potential cause of the outcome—i.e. an environmental stimulus or experience. The later phenotype might be a behaviour, physiological condition, morphology or life-history trait. The latency period between the exposure and outcome complicates causal inference due to the inevitable occurrence of additional events that may affect the relationship of interest. Here, we describe six distinct but non-mutually exclusive conceptual models from the field of lifecourse epidemiology and discuss their applications to EEB research. The models include Critical Period with No Later Modifiers, Critical Period with Later Modifiers, Accumulation of Risk with Independent Risk Exposures, Accumulation of Risk with Risk Clustering, Accumulation of Risk with Chains of Risk and Accumulation of Risk with Trigger Effect. These models, which have been widely used to test causal hypotheses regarding the early origins of adult-onset disease in humans, are directly relevant to research on developmental plasticity in EEB.

## 1. Introduction

Biology is time dependent. In humans and other animals, morphology, physiology, behaviour and life-history traits change over the course of development and in response to environmental exposures. These changes are of interest to biomedical and developmental scientists focused on human health as well as ecologists and evolutionary biologists studying ontogenetic processes in diverse species.

In the early 1900s, physiologist Stockard [1,2] showed time-dependent effects of environmental stressors during embryonic development on the later phenotypes of frogs and guinea pigs. These findings provided early evidence of developmental plasticity, the phenomenon by which the same genotype yields different phenotypes due to prior differential experiences and environmental conditions [3–5]. Despite initial intrigue, interest in developmental plasticity waned for the remainder of the twentieth century due to a focus on germ theory and eugenicist beliefs that health is inherited [6]. Nevertheless multiple experimental, clinical and epidemiological studies from approximately 1930s onward were indicative of developmental plasticity, including the work of Kermack *et al.* [7], Ravelli *et al.* [8], Forsdahl [9,10], and subsequently, through research by Barker *et al.* [11], a physician–scientist who observed inverse associations between birthweight, an indicator of the *in utero* nutritional environment, and mortality from cardiovascular disease. These findings galvanized the fetal origins of adult disease (FOAD) hypothesis, which focused on intrauterine risk factors for adult disease. FOAD gave rise to developmental origins of health and disease (DOHaD), which considers risk factors for chronic disease, not only in the fetal period, but also across all of the development [12]. N.B., the above is an overview of select historical milestones pertinent to the intersection of developmental plasticity and DOHaD; for readers seeking a more precise and comprehensive history of developmental plasticity, we recommend Gilbert & Epel [13] and Stearns [14].

The notion that early exposures and experiences shape lifelong health is important not only from a biological standpoint, but also from a disease prevention perspective: if there are vulnerable development periods during which exposures and experiences affect disease risk, then implementing interventions during these life stages could markedly improve health across the lifespan in an efficient and cost-effective manner [15]. Hand-in-hand with the rising interest in DOHaD was the recognition of challenges inherent to studying the early origins of chronic disease, chief among them being the long period between the occurrence of a risk factor and the manifestation of disease. The slow and progressive nature of chronic disease development requires extended—and ideally, frequent—data collection to link early risk factors to disease occurrence. Yet, analyses of such data are fraught with analytical challenges that hamper understanding of the sequence of events that culminate in disease. These challenges forged a parallel rise in lifecourse epidemiology, a field that specializes in analyses of data spanning years, decades, and even generations to draw causal inferences about the effect of one or more exposure on disease risk. Of particular importance are lifecourse epidemiology conceptual frameworks that organize key variables in a study system to guide study design, data analysis and interpretation of results.

Developmental plasticity is central not only to studies of human health, but also for many ecology and evolutionary biology (EEB) research programmes. As evidenced by growing literature on reaction norms and polyphenisms investigating how nutrition, predator exposure, temperature and social environment influence phenotypes in diverse taxa (reviewed in [16–18]), there is vast interest in mechanisms of developmental plasticity, the extent to which such mechanisms link experiences to phenotypes [19] and whether these phenomena are subject to constraints [20]. Further, the importance of the timing of exposures in shaping the phenotypes upon which natural selection acts is increasingly recognized [21–24]. Central to each of these inquiries is how evolution has shaped the form of developmental plasticity [5,14,25,26]. In this context, emphasis has been placed on the predictive adaptive response and the developmental constraints hypotheses. The predictive adaptive response hypothesis posits that organisms optimize their phenotype based on anticipated environmental conditions per their early exposures and experiences. The developmental constraint hypothesis posits that organisms faced with early life challenges invest in immediate survival at the cost of developmental processes that, in turn, may have long-lasting effects on health and fitness. Both hypotheses have been put forth to explain the diversity of developmental plasticity observed in nature [20,25,27].

Given that developmental plasticity shapes the phenotypes upon which selection acts [5,14,28,29], the type and timing of exposures and experiences affect phenotype both within and across generations. In this paper, we focus on within-generation effects and present lifecourse epidemiology conceptual models using examples from EEB research to highlight how these models can elucidate proximate explanations for environmentally induced phenotypic variation. A clearer understanding of the mechanistic and ontogenetic processes that contribute to developmental plasticity will shed light on existing hypotheses at the intersection of EEB and human health research, including the predictive adaptive response and constraint hypotheses [19,20,27,30,31]. However, interdisciplinary work first requires clear definitions of terms commonly used in each field. To facilitate dialogue between lifecourse epidemiologists and EEB researchers, box 1 defines common terms used throughout this paper.

Box 1. **Definitions of common terms used in lifecourse epidemiology.**

**Table:** 

term(s)	key concepts and definitions
conceptual model	A diagrammatic depiction of a hypothesis, including the temporal and causal relationships among key variables within a study system that are relevant to the research question at hand. Conceptual models typically comprise the independent variable(s) of interest, the dependent variable and additional third variables (e.g. mediators and effect modifiers).
exposure, experience, risk factor	The independent variable of interest; a potential cause of the outcome.
outcome, phenotype, disease	The dependent variable of interest, or the ultimate endpoint for which we seek to understand the series of events leading to its occurrence. This variable could be a phenotype, including morphology, e.g. form or structure; physiology, e.g. the internal mechanisms that govern function; behaviour, e.g. actions and reactions to stimuli; and life-history traits, e.g. patterns of growth and reproduction as well as longevity. This variable could also be a disease, as is often the case in human health studies.
effect modifier/effect modification	Effect modification is a phenomenon where a variable changes the direction, magnitude or significance of the relationship between an exposure and outcome. The variable responsible for this phenomenon is the effect modifier.
biological interaction	Synergy or antagonism; where the combined (i.e. joint) effect of two independent variables on the dependent variable is greater, as in the case of synergy, or smaller, as in the case of antagonism, than expected based on the sum of the effects of each independent variable alone. The distinction between biological interaction and effect modification is that for the former, the effects of both exposures are of interest, whereas for the latter, only the effect of one exposure is of interest (see [32]).
statistical interaction	The product term between two variables in a statistical model for which the *p*-value is indicative of the presence of effect modification or a biological interaction. This statistical property is related to but conceptually distinct from effect modification and biological interaction and cannot be used to differentiate between the two.
mediator/mediation	A mediator is a variable on the causal pathway between the exposure and outcome. This variable is caused by the exposure and a potential cause of the outcome. Analyses that seek to understand causal pathways through which exposures affect outcomes are referred to as mediation analyses.
direct, indirect and total effects	In mediation analysis, variance from the model can be decomposed into an indirect effect which operates through the mediator (exposure → mediator → outcome), and a direct effect that captures any remaining effect of the exposure on the outcome that does not operate through the mediator. The sum of the direct and indirect effects should equal the total effect of the exposure on the outcome, which is equivalent to the effect estimate for the exposure regressed on the outcome without accounting for any post-exposure events.
critical period	A life stage or time frame that typically begins and ends abruptly, during which external factors, e.g. exposures or experiences, have a permanent effect on future phenotype that cannot be modified by subsequent experiences. The ‘Critical Period with No Later Modifiers' model embodies this concept.
sensitive period	A life stage or time frame that typically begins and ends gradually, during which external factors (e.g. exposures or experiences) have a larger effect on future physiology or phenotype than in other periods, but the effect of the exposure can be modified by subsequent experiences. Accordingly, the ‘Critical Period with Later Modifiers’ model technically refers to a sensitive and not a critical period.

## 2. Lifecourse epidemiology conceptual models

In the early 2000s, epidemiologists Kuh & Ben-Shlomo [33,34] discussed six conceptual models that explain how one or more risk factors lead to the development of chronic disease. The conceptual models fall under the two distinct but non-mutually exclusive paradigms of ‘Critical Period’ or ‘Accumulation of Risk.’ Critical Period models focus on exposures during specific phases of development and assess their effects on future phenotype. Accumulation of Risk models consider multiple risk factors—and in some instances, the interactions among multiple risk factors—occurring either in a single life stage or across multiple life stages spanning the lifecourse. These conceptual models serve a dual purpose: (i) to illustrate the ways in which chronic disease may arise from experiences and exposures across the lifespan and (ii) serve as a guiding framework for study design and implementation of data analysis in a manner that is consistent with the investigator's hypothesis [35]. The latter will be the focus of this paper.

**Figure 1.**
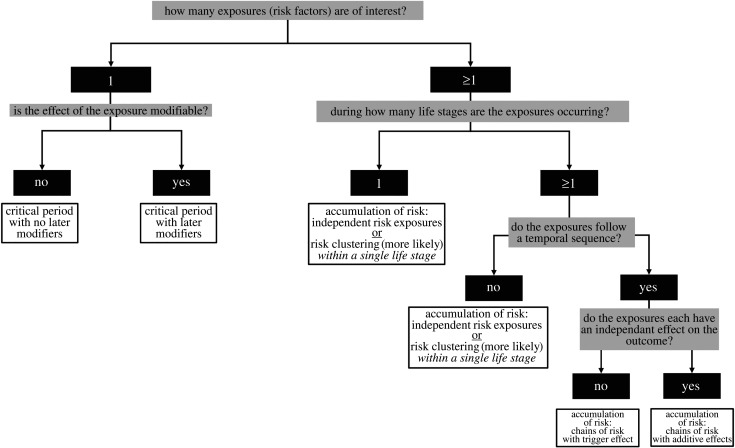
An overview and decision tree for determining which lifecourse model aligns with a research question and dataset.

Figure 1 provides an overview of when each conceptual model is most relevant based on the number of exposures (risk factors) of interest, when they occur and whether they interact with other exposures. It is important to note that these conceptual models are general frameworks that may be tailored for specific research questions. Moreover, as biology rarely fits into discrete categories, it is often the case that more than one lifecourse model may apply to a given phenomenon. This does not negate the utility of the lifecourse framework because, unlike biology, analytical models and approaches require simplifying assumptions and a clear analytical plan is foundational to advancing understanding of complex phenomena. Use of conceptual models can also help to identify the life stages and exposures/experiences that explain the observed variation in a phenotype (or disease) of interest, laying the foundation for future targeted research.

## 3. Applications of lifecourse epidemiology conceptual models in ecology and evolutionary biology

Here, we present the six lifecourse epidemiology conceptual models, as named and described by Ben-Shlomo & Kuh [33,34], and applications of each model using examples from EEB. With adequate prior knowledge, investigators may select a conceptual model that best explains the phenomenon at hand and use the model to guide data analysis. This approach leverages the conceptual model in an evidence-based manner to drive the analytical approach (figure 1). Alternatively, investigators may test one or more conceptual models empirically in order to determine which (if any) best represents developmental processes that culminate in the outcome of interest. In this scenario, investigators may not have a hypothesis about which conceptual model best explains the relationship of interest; rather, they seek to determine the accuracy or relevance of one or more models given the data. Data-driven approaches exist for identifying which lifecourse model best fits the data [36], but are not the focus of this paper.

In the following sections, we share examples of each conceptual model from the Mara Hyena Project, a long-term field study of wild spotted hyaenas (*Crocuta crocuta*) in the Masai Mara National Reserve, Kenya. With over 30 years of social, environmental and physiological data collected from greater than 4000 individually identifiable hyaenas across six generations [37], this study system provides a parallel to long-term human cohorts used to study determinants of chronic disease. In addition to the suitable longitudinal data, spotted hyaenas exhibit protracted development with clearly defined life-history stages and live relatively long lives [38,39]. The life history of this species, coupled with extensive prior research on their development, provides tractable examples for lifecourse analyses. While other natural systems also serve as excellent examples for the study of developmental plasticity (electronic supplementary material, table S1), we focus on examples from hyaenas in the main text to leverage our biological expertise.

### (a) Critical Period models

#### (i) Critical Period with No Later Modifiers

The Critical Period with No Later Modifiers model posits that an exposure that occurs during a specific, well-defined life stage has a long-lasting effect on phenotype (e.g. a morphological feature, a physiological condition, a behavioural trait, subclinical or clinical disease) that is not altered by subsequent exposures. This model indicates a deterministic and sufficient effect of the exposure on an organism's future phenotype.

**Figure 2.**
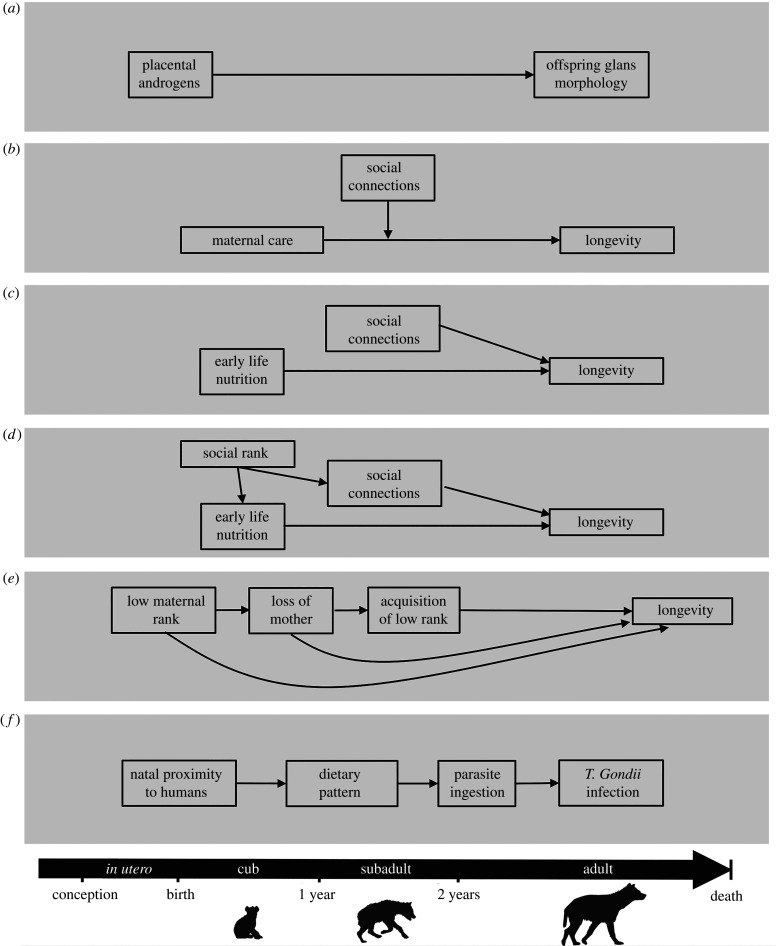
Lifecourse conceptual models using examples from spotted hyenas. (*a*) Critical Period with No Later Modifiers. (*b*) Critical Period with Later Modifiers. (*c*) Accumulation of Risk with Independent Risk Exposures. (*d*) Accumulation of Risk with Risk Clustering. (*e*) Accumulation of Risk: Chains of Risk with Additive Effects. (*f*) Accumulation of Risk: Chains of Risk with Trigger Effects.

In spotted hyaenas, *in utero* exposure to androgenic hormones causes sex differences in the development of the morphology of the glans of the phallus. The biology underlying this phenomenon results from the feminizing effects of fetal treatment with anti-androgens [40,41]. Because there is evidence to suggest the irreversibility of the organizational process through which androgens affect glans morphology [42,43], we use this as an example to illustrate the Critical Period with No Later Modifiers model (figure 2*a*).

With adequate prior knowledge that *in utero* exposure to elevated androgens has organizational effects on glans morphology, investigators can conduct the analysis without considering post-exposure events (i.e. anything that happens after the gestation period), though they should account for confounders, or shared common causes of the exposure and outcome that introduce bias into the estimate of association (see [44]). Following data analysis, the interpretation of results should focus on the effect estimate only for the exposure of interest, which in this example represents the effect of prenatal androgen exposure on adult glans shape.

In the absence of adequate literature supporting this conceptual model (or, in pursuit of alternative explanations for a phenomenon), investigators may test whether this model adequately depicts the effect of prenatal androgen exposure on glans morphology. This approach can involve running a series of models, starting first by investigating the relationship between androgen exposure *in utero* and glans shape in adulthood, then accounting for exposures occurring after birth that might subsequently influence glans morphology. If the inclusion of later exposures as covariates in the model attenuates the effect estimate for prenatal androgen exposure towards the null, then the relationship of interest does not follow the Critical Periods with No Later Modifiers paradigm. At this point, investigators may report results, or test a different conceptual model.

#### (ii) Critical Period with Later Modifiers

The Critical Period with Later Modifiers model posits that the effect of an exposure on an outcome can be altered by events that occur after the initial exposure (figure 1*b*). Before exploring this model, we and others make a distinction between ‘critical’ and ‘sensitive’ periods (box 1; see also [22,23]). Both terms refer to timeframes characterized by high developmental plasticity, making it such that external stimuli during these periods have a larger effect on future phenotype than those occurring at other life stages. However, a critical period typically begins and ends abruptly, occurs very early in the lifecourse, and is a timeframe in which a specific ontogenetic process must occur to ensure normal development. On the other hand, a sensitive period tends to begin and end more gradually and is a time when a particular developmental process is most likely to occur. Accordingly, it can generally be stated that the effect of an exposure during a critical period is irreversible (and thus appropriate for the scenario described in 3a(i)), while some degree of future enhancement or recovery following the initial exposure is possible if an exposure occurs during a sensitive period. Therefore, the Critical Period with Later Modifiers model is somewhat of a misnomer as it more likely refers to a sensitive period, though we refer to it as a critical period in the remainder of this paper for consistency with the literature.

The Critical Period with Later Modifiers model aligns with the concept of effect modification, where the effect of the exposure on the outcome varies with respect to a third variable, which is the ‘later modifier.’ In spotted hyaenas, the infancy and the subadult life stages appear to be critical periods in which positive social experiences promote longevity. Indeed, offspring who receive better maternal care live longer than peers receiving inferior maternal care [45]. Having a larger number of social connections with group mates during the subadult life stage is also associated with longevity [46]. Therefore, later-life social connections may modify (i.e. change the nature of the association) the beneficial effect of maternal care on longevity. In this scenario, our research question centres on whether the effect of early life maternal care on longevity varies by social connectedness during the subadult life stage (figure 2*b*).

If there is prior literature to support the relevance of this conceptual model to the phenomenon of interest, then investigators may implement stratified analysis from the start. This entails running models for the relationship between maternal care and longevity within strata of the effect modifier, social connectedness during subadulthood (e.g. high versus low). If the stratum-specific estimates for the exposure of interest differ in magnitude, direction and/or precision, then such results support this conceptual model. Prior to stratified analyses, investigators often test for a statistical interaction between the exposure (maternal care) and effect modifier (high versus low number of social connections during the subadult life stage). If the *p*-value for the product term falls below the threshold of a pre-specified alpha, then this provides evidence of effect modification and supports the decision to conduct stratified analysis. If the product term is not significant, then this suggests that Critical Period with Later Modifiers is not the appropriate conceptual model for this research question, and investigators can report the main effect for the association of interest (i.e. the effect of maternal care on longevity, for all hyaenas, regardless of social connectedness during subadulthood, obtained from a model without an interaction term).

When assessing Critical Period with Later Modifiers, the later modifier may also be a causal mediator, or a variable that is affected by the exposure and a possible determinant of the outcome (box 1). Researchers should not confuse the concept of effect modification with mediation. ‘Effect modification’ refers to heterogeneity in the effect of the exposure on the outcome across levels of a third variable, whereas ‘mediation’ describes a scenario when the effect of the exposure operates at least in part through a third variable that lies on the causal pathway [44]. Investigators may be tempted to use regression-based analysis to compare the effect estimate for the exposure before versus after adjusting for the later modifier to test this conceptual model [47]. However, this approach addresses a fundamentally different question that revolves around causal pathways through which the exposure may affect the outcome, as opposed to whether the effect of the exposure on the outcome varies with levels of the modifier.

Returning to our hyaena example, social connections during the subadult life stage may be a mediator, in addition to an effect modifier since a hyaena's social connectedness in subadulthood could be affected by the quality of mother–offspring interactions during early life [45]. If this is the case, simple stratification by subadult social connectedness may introduce bias into the effect estimate for maternal care due to the potential for unmeasured confounders to the relationship between social connectedness during subadulthood (mediator) and longevity (outcome) [44]. In such a scenario, analytical approaches such as the counterfactual approach, which allows for statistical interaction between the exposure and effect modifier [48], or non-parametric methods such as inverse probability weighting [49] allow for interdependency between the exposure and effect modifier.

#### (iii) Additional considerations for the Critical Period models

When considering multiple critical and/or sensitive periods simultaneously, investigators may be interested in assessing whether the occurrence of the exposure of interest during a specific life stage has a larger impact on phenotype than if that exposure were to occur during a different life stage. This can be accomplished using the conditional standardized residual approach, which entails deriving residuals at each life stage conditional on prior life stages and including conditional residuals for all age periods in a single regression model [50]. Although it is more realistic to consider multiple critical or sensitive periods given the complexity of adult phenotypes, this is not always feasible due to constraints of data availability and/or statistical power.

### (b) Accumulation of Risk models

#### (i) Accumulation of Risk with Independent Risk Exposures

The Accumulation of Risk with Independent Risk Exposures model describes a scenario where an individual is exposed to multiple, unrelated risk factors that are experienced concurrently (i.e. in the same life stage) or asynchronously (i.e. across multiple life stages). In spotted hyaenas, both access to food and social support influence longevity, but an individual's social support during the subadult or adult life stages cannot affect access to food during the perinatal period and therefore, represents an independent and unrelated risk factor. Early life nutrition is critical to hyaena development as suggested by more conceptions occurring during periods of high than low seasonal food abundance [51], and the association between juvenile mass, a proxy for food intake and survival [52]. As discussed above, subadults with greater social connectivity live longer [46], an effect that likely operates through pathways unrelated to early life nutrition (e.g. current social support or resource access), rendering the two as unrelated and independent determinants of longevity. Using the Accumulation of Risk with Independent Risk Exposures, we ask if early life nutrition and offspring social connections are each independent determinants of longevity in hyaenas (figure 2*c*).

To assess this model, investigators must conduct two sets of analyses. The first analysis entails running separate regression models to estimate the effect of each exposure on the outcome (unadjusted models). The second analysis involves running a model in which all exposures of interest are included in the same model (multiple variable model). If Accumulation of Risk with Independent Risk Exposures is a suitable conceptual model for the outcome of the study, we can expect to observe similar effect estimates for each risk factor based on the unadjusted models relative to those effect estimates obtained from the multiple variable model. The similar effect estimates from the unadjusted versus multiple variable models results from the uncorrelated nature of the risk factors, and the independent effects of each on longevity. If the estimates in unadjusted and adjusted models are similar, then there is evidence to support this model. If they are different, then it is likely that the risk factors are correlated, potentially via the Accumulation of Risk with Risk Clustering phenomenon, discussed below.

#### (ii) Accumulation of Risk with Risk Clustering

Accumulation of Risk with Risk Clustering is the alternative scenario to what we described in 3b(i). Here, risk factors that appear unrelated may co-occur because of a shared common cause. Building on our previous example, one such common cause of early life nutrition and later-life social connectedness in hyaenas is social dominance rank—a determinant of access to resources, including food [53–55] and a determinant of social connections as hyaenas often choose to associate with groupmates based on their social rank [56].

There are numerous ways in which this model may guide analyses, depending on the research question. One question stemming from this conceptual model could revolve around understanding whether one particular pathway linking social rank to longevity is more important than others (early life nutrition or social connectivity). Such a question would entail a mediation analysis using non-parametric approaches like inverse probability weighting [57–59] to ascertain and compare path-specific effects of social rank on longevity operating through early life nutrition and social connectedness (figure 2*d*).

If investigators seek to test the appropriateness of this conceptual model, clustering algorithms can be used to group individuals based on the proximal exposures of interest (early life nutrition and social connectivity), followed by an assessment of the extent to which membership in distinct groups varies with social rank in a manner consistent with the hypothesis—e.g. the group characterized by favourable early life nutrition and high social connectivity in subadulthood also has a higher proportion of individuals with high social rank, and vice versa for a group with poor early life nutrition and low social connectivity.

#### (iii) Accumulation of Risk: Chains of Risk with Additive Effects

In lifecourse studies, researchers are often interested in the cumulative effects of multiple exposures across developmental stages given that this scenario is likely a common occurrence. This objective aligns with the Chains of Risk with Additive Effects model, in which multiple exposures contribute to disease risk. Here, each exposure increases the likelihood of subsequent exposures, and each exposure not only has a direct effect on the outcome, but also has an indirect effect through later exposures (box 1). The exposures interact in a synergistic fashion where two or more exposures, in combination, have a larger effect on the outcome than the sum of each individual exposure by itself—a phenomenon known as biological interaction [32].

In diverse species, multiple adverse early life experiences have cumulative detrimental effects on longevity [60]. In spotted hyaenas, offspring born to low-ranking mothers and offspring whose mothers die before offspring reach adulthood both have reduced longevity [61]. The process of rank acquisition also presents a form of adversity associated with reduced longevity [62]. Not only are each these early adverse experiences independently associated with reduced longevity, but it is also highly plausible that these variables affect one another in a sequential temporal fashion such that individuals exposed to all three adverse early life experiences will exhibit markedly reduced longevity than those who experience fewer adverse experiences (figure 2*e*).

One approach to analysing data in alignment with this conceptual model includes mediation analyses assessing various pathways through which the exposures can influence the outcome, including causal path analyses to estimate path-specific effects of sequential events [63]. If investigators are uncertain about the sequence of events, there are data-driven generalized causal mediation methods that allow for multiple mediators and that make no assumptions about the causal ordering among the mediators occurring during the same stage [64]. However, this approach does not allow for explicit assessment of additive effects (*synergy*) among the multiple exposures.

Thus, a more appropriate approach is to use the count of adverse experiences as the exposure variable of interest. This allows for the estimation of the combined effects of an increasing number of risk factors on longevity. If a higher count of adverse experiences has a larger effect on decreased longevity than would be expected based on the sum of the effect for each individual exposure, then this provides evidence for chains of risk with additive effects. This has been implemented in hyaenas using the variables shown in figure 2*e* [62]. A similar approach has been employed in wild baboons [65]. The primary limitation to this approach is that it assumes the same magnitude of effect for all risk factors, which is unlikely to be the case given that some adverse experiences will have a larger impact on future phenotype than others. Additionally, this method does not specifically test the possibility that the occurrence of one exposure increases the likelihood of occurrence of subsequent exposures, and therefore this analytical strategy could also be used to test the Accumulation of Risk with Independent Risk Exposures model. In analyses where investigators seek to determine whether their data follow Accumulation of Risk with Independent Risk Exposures versus Accumulation of Risk with Additive Effects, a key feature of the latter is evidence of biological interaction among multiple exposures. It is important to note, however, that biological interactions include both *synergy* as well as *antagonism*, where the effect of one exposure cancels out the effect of another exposure, making it such that the combined effect of the two exposures together is smaller than the sum of each individually. To this end, conceptual models are also helpful for identifying explanations for null results when the literature suggests otherwise—e.g. there may be antagonism caused by an unmeasured variable.

Of note, a key distinguishing feature between Critical Period with Later Modifiers and Accumulation of Risk with Additive Effects is that in the former, one specific exposure is of interest—i.e. the exposure occurring during the ‘critical period.’ The ‘later modifier’ is a third variable for which investigators may assess the effect of the exposure on the outcome within strata of that variable, or for which investigators may want to condition on in mediation analysis, without interpreting its coefficient (discussed in 3a(ii)). On the other hand, in Accumulation of Risk with Additive Effects, the effect of multiple exposures are of interest—both in combination, and each alone, in order to determine whether a biological interaction is present. This distinction coincides with the distinction between effect modification and biological interaction, discussed in detail elsewhere [32].

#### (iv) Accumulation of Risk: Chains of Risk with Trigger Effect

Chains of Risk with Trigger Effect posits that each exposure in a time-ordered sequence leads to subsequent exposures, but only the final exposure causes (triggers) the outcome to occur. While many biological processes follow this model, it can be challenging to identify lifecourse phenomena that reflect this model given that organisms often express phenotypes gradually in response to multiple exposures. However, the contraction of infections represents a tractable example for this conceptual model (figure 2*f*).

Spotted hyaenas exhibit a high prevalence of infection with the parasite *Toxoplasma gondii* [66,67]. A doubling of infection in older hyaenas suggests that the parasite is transmitted via the consumption of infected meat [66]. A recent meta-analysis reported that proximity to human populations is a risk factor for *T. gondii* infection due to higher exposure to cats, the definitive host of the parasite [68]. Based on these data we may hypothesize a causal relationship between risk factors and infection with *T. gondii* that follows the Chains of Risk with Trigger Effect paradigm. In spotted hyaenas, being born near pastoralist communities may be viewed as the initial risk factor. Subsequently, proximity to these human communities may lead to consumption of infected domestic livestock, and ultimately, ingestion of a *T. gondii* infected animal. While each of these risk factors increases the probability of infection, the final trigger is ingestion of the parasite.

Whether the analysis is driven by prior knowledge or seeks to test the relevance of this model to the phenomenon at hand, an analytical approach that aligns with this model is to examine the association between each link in the chain and the outcome before versus after conditioning on a subsequent event. If there is only one pathway leading to the outcome, then conditioning on any subsequent event by statistical adjustment, weighting or stratification will attenuate the effect of the association to the null. If this is not the case, then Accumulation of Risk with Additive Effects may be more appropriate.

#### (v) Additional considerations for the Accumulation of Risk models

Unlike the Critical Period models, the Accumulation of Risk models involve multiple pathways linking exposures to the outcome. Such complexity lends flexibility to the analytical approach, as is evident in our suggestions of multiple analytical strategies for a given conceptual model. However, this flexibility is a double-edged sword: although it allows for creativity in regard to research questions and data analyses, it also often results in mis-parameterized models yielding estimates of association that no longer answer the original research question. Therefore, clearly defining the research question and the specific effect of interest represented by paths in the conceptual model is paramount.

In 2016, Ben-Shlomo & Kuh [33,34] revisited the conceptual models described in their original papers and discussed the utility of lifecourse epidemiological frameworks for research on ageing [69]. In this later publication, they acknowledged that it is more sensible to view critical or sensitive period models as sub-sets of an accumulation of risk model when considering the effects of an exposure over time. Although we do not go into the cumulative effects of a single exposure across the lifecourse (e.g. the cumulative effect of social stress over time), we acknowledge this phenomenon is relevant and there are advanced methods appropriate for this type of research question [70]. However, for clarity and illustrative purposes, we have described each Accumulation of Risk model using distinct exposures.

## 4. Conclusion

As heuristic tools used to generalize and organize patterns and processes observed in nature, lifecourse epidemiology conceptual models help investigators develop and test hypotheses using empirical data [35]. Although conceptual frameworks are widely used in EEB to investigate ecological and evolutionary processes (see [71–75]), a unified conceptual framework for the study of developmental plasticity across ontogeny is lacking. Lifecourse epidemiology conceptual models have proven utility and value in understanding risk factors, mechanisms and aetiological pathways underlying the development of chronic disease in humans—an endpoint that shares common hallmarks with the study of phenotypes that arise from the process of developmental plasticity in populations of wild animals.

Similar to how lighthouses illuminate certain parts of the ocean and adjacent shoreline (the exposure, outcome and key third variables) while leaving other parts in the dark (variables outside of the study system) [76], conceptual models bring focus to specific aspects of a research question to guide the analysis in an efficient and effective manner. Ultimately, it is our hope that the concepts discussed herein encourage the application of the lifecourse framework in diverse systems—both free-living populations, as well as laboratory studies of developmental plasticity.

## Data Availability

No empirical data were collected for this project.
